# Ischemic Tolerance Protects the Rat Retina from Glaucomatous Damage

**DOI:** 10.1371/journal.pone.0023763

**Published:** 2011-08-24

**Authors:** Nicolás Belforte, Pablo H. Sande, Nuria de Zavalía, Diego C. Fernandez, Dafne M. Silberman, Mónica S. Chianelli, Ruth E. Rosenstein

**Affiliations:** 1 Laboratory of Retinal Neurochemistry and Experimental Ophthalmology, Department of Human Biochemistry, School of Medicine, University of Buenos Aires/CEFyBO, CONICET, Buenos Aires, Argentina; 2 Laboratory of Histology, School of Medicine, University of Morón, Pcia de Buenos Aires, Argentina; The University of Hong Kong, Hong Kong

## Abstract

Glaucoma is a leading cause of acquired blindness which may involve an ischemic-like insult to retinal ganglion cells and optic nerve head. We investigated the effect of a weekly application of brief ischemia pulses (ischemic conditioning) on the rat retinal damage induced by experimental glaucoma. Glaucoma was induced by weekly injections of chondroitin sulfate (CS) in the rat eye anterior chamber. Retinal ischemia was induced by increasing intraocular pressure to 120 mmHg for 5 min; this maneuver started after 6 weekly injections of vehicle or CS and was weekly repeated in one eye, while the contralateral eye was submitted to a sham procedure. Glaucoma was evaluated in terms of: i) intraocular pressure (IOP), ii) retinal function (electroretinogram (ERG)), iii) visual pathway function (visual evoked potentials, (VEPs)) iv) histology of the retina and optic nerve head. Retinal thiobarbituric acid substances levels were assessed as an index of lipid peroxidation. Ischemic conditioning significantly preserved ERG, VEPs, as well as retinal and optic nerve head structure from glaucomatous damage, without changes in IOP. Moreover, ischemia pulses abrogated the increase in lipid peroxidation induced by experimental glaucoma. These results indicate that induction of ischemic tolerance could constitute a fertile avenue for the development of new therapeutic strategies in glaucoma treatment.

## Introduction

Glaucoma is a leading cause of blindness worldwide, characterized by specific visual field defects due to the loss of retinal ganglion cells (RGCs) and damage to the optic nerve head (ONH). The result is a patchy loss of vision, generally in a peripheral to central manner. It is estimated that half of those affected may not be aware of their condition because symptoms may not occur during the early stages of the disease. When vision loss appears, considerable permanent damage has already occurred. Medications and surgery can help to slow the progression of some forms of the disease, but there is no cure at present.

Unraveling which are the most critical mechanisms involved in glaucoma is unlikely to be achieved in studies which are limited to the clinically observable changes to the retina and optic nerve head that are seen in human glaucoma. Far more detailed and invasive studies are required, preferably in a readily available animal model. Recently, we have developed a model of glaucoma in rats through weekly injections of chondrotin sulfate (CS) in the eye anterior chamber. Acute or chronic intracameral injections of CS significantly increase IOP as compared with vehicle-injected eyes [1]. Moreover, injections of CS for 6 or 10 (but not 3) weeks significantly decrease the electroretinographic activity as well as flash visual evoked potentials (VEPs). After 10 weeks of ocular hypertension induced by CS, a significant loss of ganglion cell layer (GCL) cells and optic nerve fibers occurs in eyes treated with CS [1]. These results indicate that weekly intracameral injections of CS mimic central features of human primary open-angle glaucoma. Thus, this model could be a useful tool for understanding the pathogenic mechanisms involved in glaucomatous neuropathy, as well as for the development of new therapeutic strategies.

The major risk factor for glaucoma is the increased intraocular pressure (IOP), and its pharmacological and/or surgical reduction slows down the progression of glaucomatous damage. However, lowering ocular hypertension does not completely stop damage progression, indicating risk factors other than IOP. It has been consistently suggested that an elevation of IOP evokes a variety of consequential events, including reduction in blood flow which leads to a partial ischemic insult [2], [3]. In that sense, several evidences support a localized vascular insufficiency leading to perfusion deficits of ocular structures, including the ONH, the retina, the choroid, and the retrobulbar vessels [4]. Combined with high IOP, ischemic mechanisms can cause oxidative stress, reperfusion damage, and ultimately axon loss [5]. Several animal and human studies have indicated that vascular dysregulation and ischemia play a role in glaucoma pathogenesis [6]–[9]. Retinal ischemia develops when retinal blood flow is insufficient to match the metabolic needs of the retina, one of the highest oxygen-consuming tissues. Ischemia impairs retinal energy metabolism, and triggers a reaction cascade which can result in cell death. Oxidative stress, excitotoxicity, calcium influx, and others mechanisms acting in tandem are of considerable importance in retinal ischemic damage (reviewed in [10]). Notably, most of these mechanisms are also involved in glaucomatous neuropathy [11], [12]. Although there is no effective treatment against retinal ischemic injury, it is possible to activate an endogenous protection mechanism by ischemic preconditioning (IPC) [13], [14]. IPC requires a brief period of ischemia applied before ischemic injury, which does not produce any significant damage *per se*, and induces tolerance to the subsequent severely damaging ischemic event (reviewed in [15]). In fact, it has been shown that IPC affords the retina a robust functional protection against ischemic damage [13]. Although IPC confers a highly significant neuroprotection in different *in vitro* and *in vivo* models of ischemia, its utilization as a clinical strategy is mostly limited because the onset of retinal ischemia is largely unpredictable, in contrast to the onset of reperfusion that could be more predictable. In this vein, another endogenous form of ischemic protection, in which a short series of repetitive cycles of brief ischemia/reperfusion (I/R) are applied immediately at the onset of reperfusion, termed postconditioning (PostC), has been reported in several tissues [16], [17]. Recently, we have shown that a 7-min pulse of ischemia applied 5 min after the reperfusion onset, induces an almost complete histological and functional protection in eyes exposed to ischemic injury [18]. Based on the highly effective protection induced by IPC and PostC against an acute ischemic episode, the aim of this work was to analyze the effect of brief ischemia pulses on retinal damage induced by experimental glaucoma.

## Results

Table 1 depicts the average IOP of rats weekly injected with vehicle or CS submitted to ischemia pulses or sham procedure at 6, 7, 8, 9, and 10 weeks of intracameral injections of vehicle or CS. IOP was significantly higher in CS- than in vehicle-injected eyes, whereas ischemia pulses did not modify IOP in both groups at all the time points examined.

**Table 1:** Effect of ischemia pulses on IOP in vehicle and CS-injected eyes.

	Intraocular pressure (mmHg)			
	vehicle		CS	
	sham	pulses	sham	pulses
6 weeks	11.30±0.5	12.1±0.8	22.1±1.1**	21.4±1.6**
7 weeks	11.8±0.8	11.8±0.6	24.6±1.5**	22.3±1.5**
8 weeks	12.0±0.6	11.9±0.7	23.4±1.3**	24.6±1.0**
9 weeks	11.7±0.8	12.1±0.9	24.8±1.6**	23.9±1.5**
10 weeks	11.9±0.6	11.5±0.7	22.0±1.0**	21.4±1.5**

TonoPen measurements of IOP from eyes bilaterally injected with vehicle or CS and submitted to ischemia pulses or sham procedure. IOP was assessed at 6, 7, 8, 9, and 10 weeks of weekly intracameral injections. At all time points examined, CS significantly increased IOP as compared with vehicle-injected eyes. Ischemia pulses did not modify this parameter in vehicle or CS-injected eyes at any time point. Data are the mean ± SEM (n = 10 animals per group).

**p<0.01 versus vehicle-injected eyes without ischemia pulses (sham), by Tukey's test.

**Figure 1:**
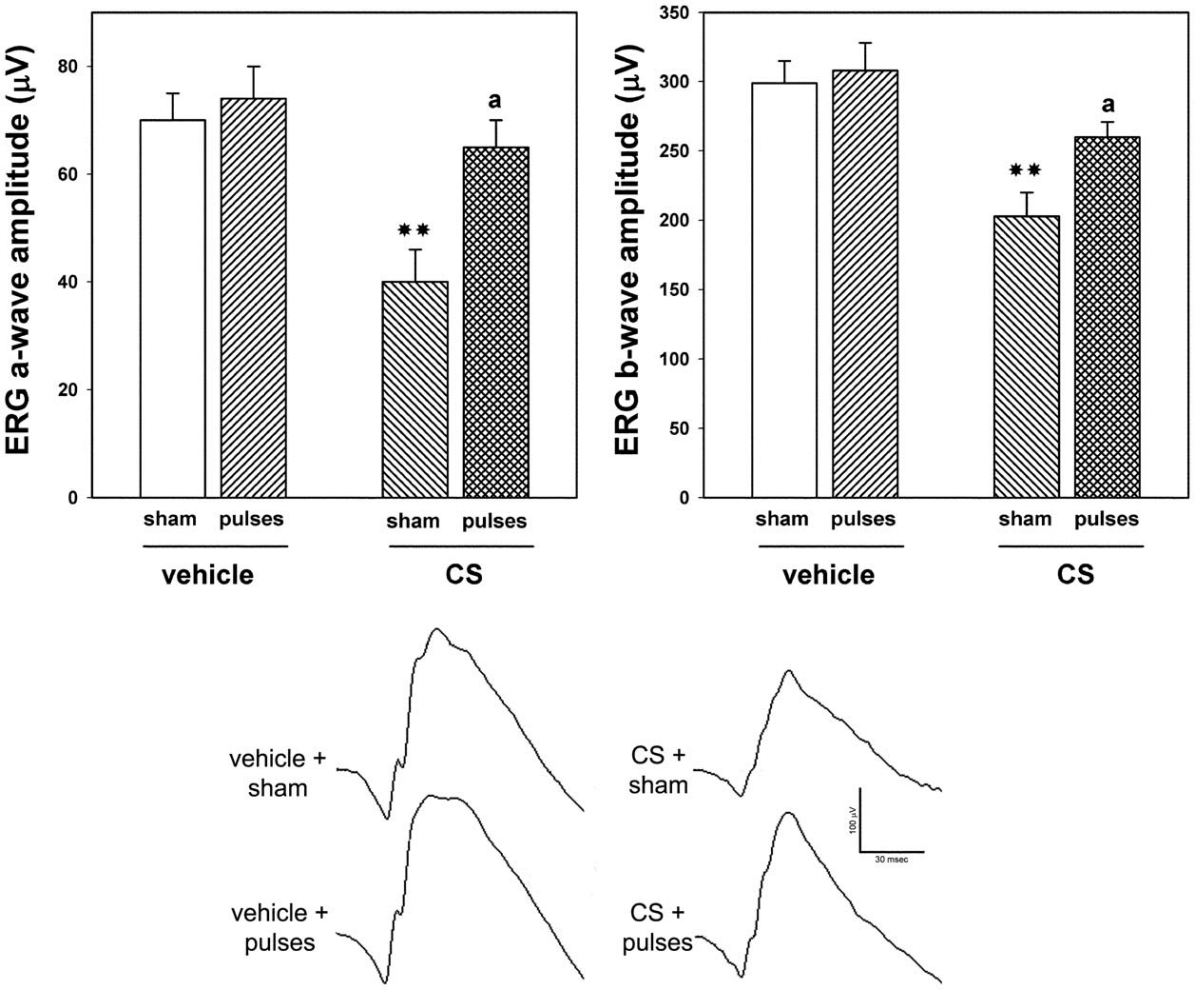
Electroretinographic preservation in hypertensive eyes induced by the application of brief ischemia pulses. ERGs were registered after 10 weeks of treatment with vehicle or CS. CS induced a significant decrease in ERG a- and b-wave amplitude, as compared with vehicle-injected eyes. In hypertensive eyes submitted to ischemia pulses, a significant reversion of these alterations was observed. The lower panel shows representative scotopic ERG traces from eyes injected with vehicle or CS without or with ischemia pulses. Data are the mean ± SEM (n = 10 animals per group); **p<0.01 versus vehicle injected eyes without ischemia pulses (sham); a: p<0.05, versus CS-injected eyes without ischemia pulses (sham), by Tukey's test.

**Figure 2:**
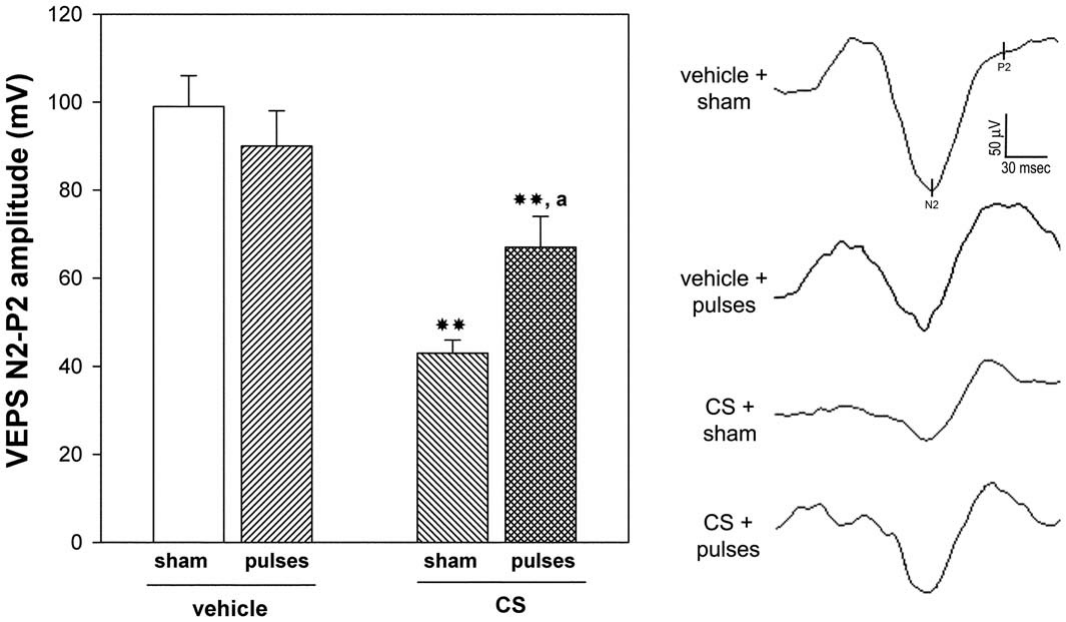
Flash VEPs in eyes injected with vehicle or CS without or with ischemia pulses. Animals were weekly injected with vehicle or CS for 10 weeks. Ischemia pulses were applied in one eye, while the contralateral eye was submitted to a sham procedure. Left panel shows average amplitudes of VEP N2-P2 component amplitude and right panel shows representative VEP traces. A significant reduction in flash VEP N2-P2 amplitude component was observed in eyes injected with CS for 10 weeks without ischemia pulses. The application of weekly ischemia pulses significantly abrogated the effect of ocular hypertension. No changes between vehicle injected eyes without or with ischemia pulses were observed. Data are mean ± SEM (n = 10 eyes/group), **p<0.01 versus vehicle injected eyes without ischemia pulses (sham), a: p<0.05 versus CS-injected eyes without ischemia pulses (sham), by Tukey's test.

In order to assess the effect of ischemia pulses on functional alterations induced by ocular hypertension, the functional state of retinas from eyes weekly injected with vehicle or CS for 10 weeks without or with ischemia pulses was analyzed by scotopic electroretinography. The average amplitudes of scotopic ERG a- and b- waves of rats bilaterally injected with vehicle or CS for 10 weeks and submitted to weekly ischemia pulses in one eye and a sham procedure in the contralateral eye are depicted in Figure 1. These parameters were significantly reduced in eyes which received CS with sham procedure as compared with vehicle-injected eyes. The application of ischemia pulses significantly abrogated the ocular hypertension-induced decrease in ERG a- and b-wave amplitude. Representative scotopic ERG traces from all the groups are shown in the lower panel of Figure 1. To assess the visual pathway function, flash VEPs were registered at 10 weeks of ocular hypertension induced by weekly injections of CS. A significant decrease in the VEP N2-P2 component amplitude was observed in eyes injected with CS without ischemia pulses as compared with vehicle-injected eyes, whereas a significant preservation of the VEP N2-P2 component was observed in hypertensive eyes submitted to ischemia pulses (Figure 2). No noteworthy differences in the VEP N2-P2 component latency were observed among groups (data not shown). Representative waveforms of VEPs for all groups are shown in the right panel of Figure 2. ERGs and VEPs in vehicle-injected eyes submitted to a sham procedure did not differ from those of vehicle-injected eyes submitted to ischemia pulses.

**Figure 3:**
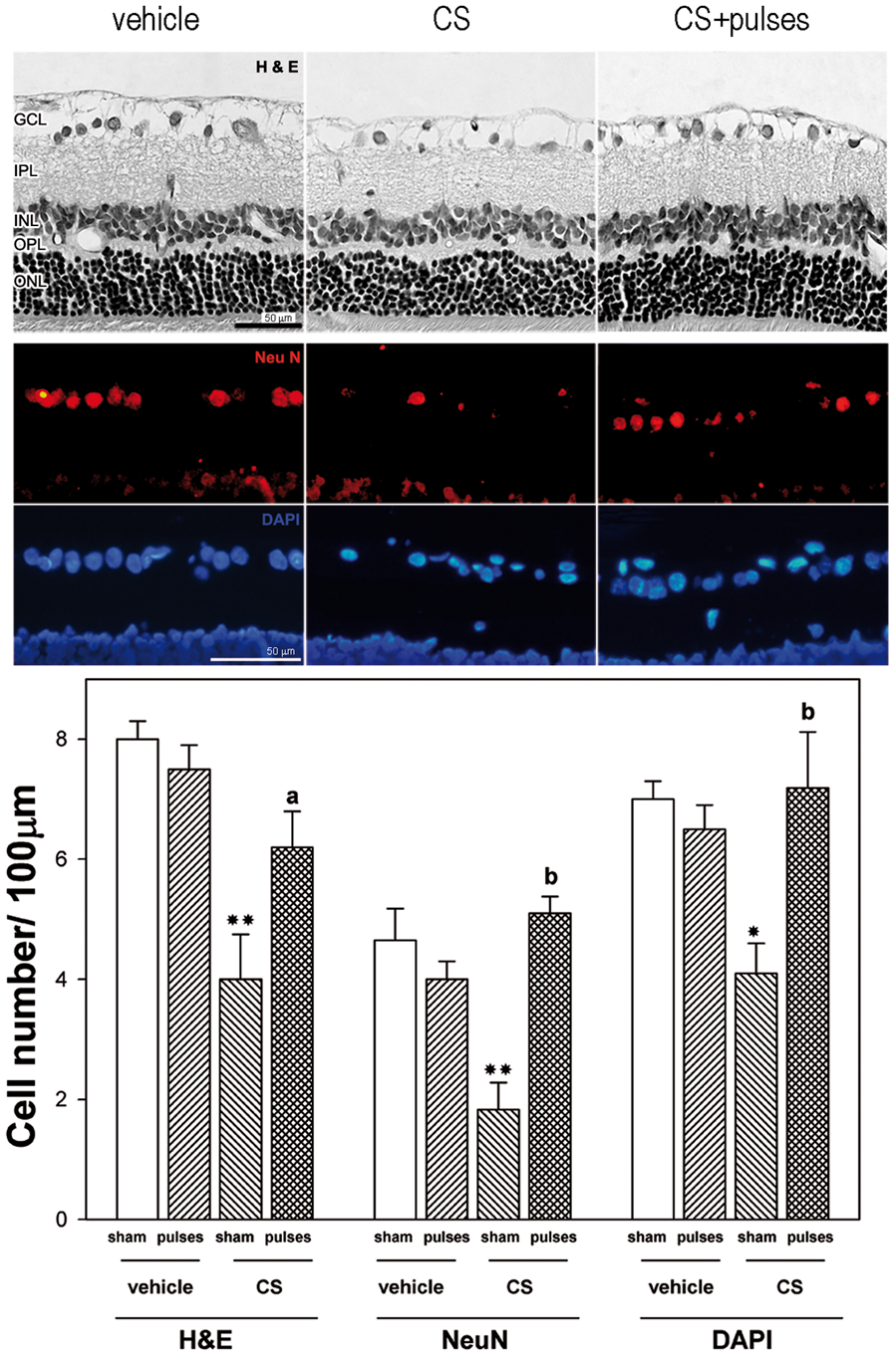
Retinal histology examination after 10 weeks of ocular hypertension. Upper panel: Representative photomicrographs of retinal sections stained with hematoxylin and eosin from a vehicle-injected eye, and a hypertensive eye without or with pulses of ischemia. Note the diminution of GCL cells in the eye injected with CS without ischemia pulses. The application of ischemia pulses preserved this parameter. The other retinal layers showed a normal appearance in all groups. Middle panel: Immunohistochemical detection of NeuN-positive neurons in the GCL from a vehicle-injected eye, a hypertensive eye without or with ischemia pulses. A strong NeuN-immunostaining (red) was confined to ganglion cells in the GCL. The number of NeuN positive ganglion cells was lower in hypertensive eyes without ischemia pulses than in vehicle- injected eyes, whereas the application of ischemia pulses in CS-injected eyes increased NeuN-immunostaining. A similar profile was observed for cell nuclei counterstained with DAPI (blue). Lower Panel: cell count in the GCL evaluated by H&E staining, NeuN immunostaining, and DAPI labeling. By all these methods, a significant decrease of the number of cells in the GCL was observed in CS- injected eyes without ischemia pulses as compared with vehicle-injected eyes (sham), whereas ischemia pulses significantly preserved this parameter in CS-injected eyes. Scale bar: Upper panel  =  50 µm; Middle panel  =  50 µm. Data are the mean ± SEM (n = 5 animals per group). * p<0.05, ** p<0.01 vehicle injected eyes without ischemia pulses (sham), a: p<0.05, b: p<0.01 versus CS-injected eyes without ischemia pulses (sham), by Tukey's test. GCL, ganglion cell layer; IPL, inner plexiform layer; INL, inner nuclear layer; OPL, outer plexiform layer; ONL, outer nuclear layer.

**Figure 4:**
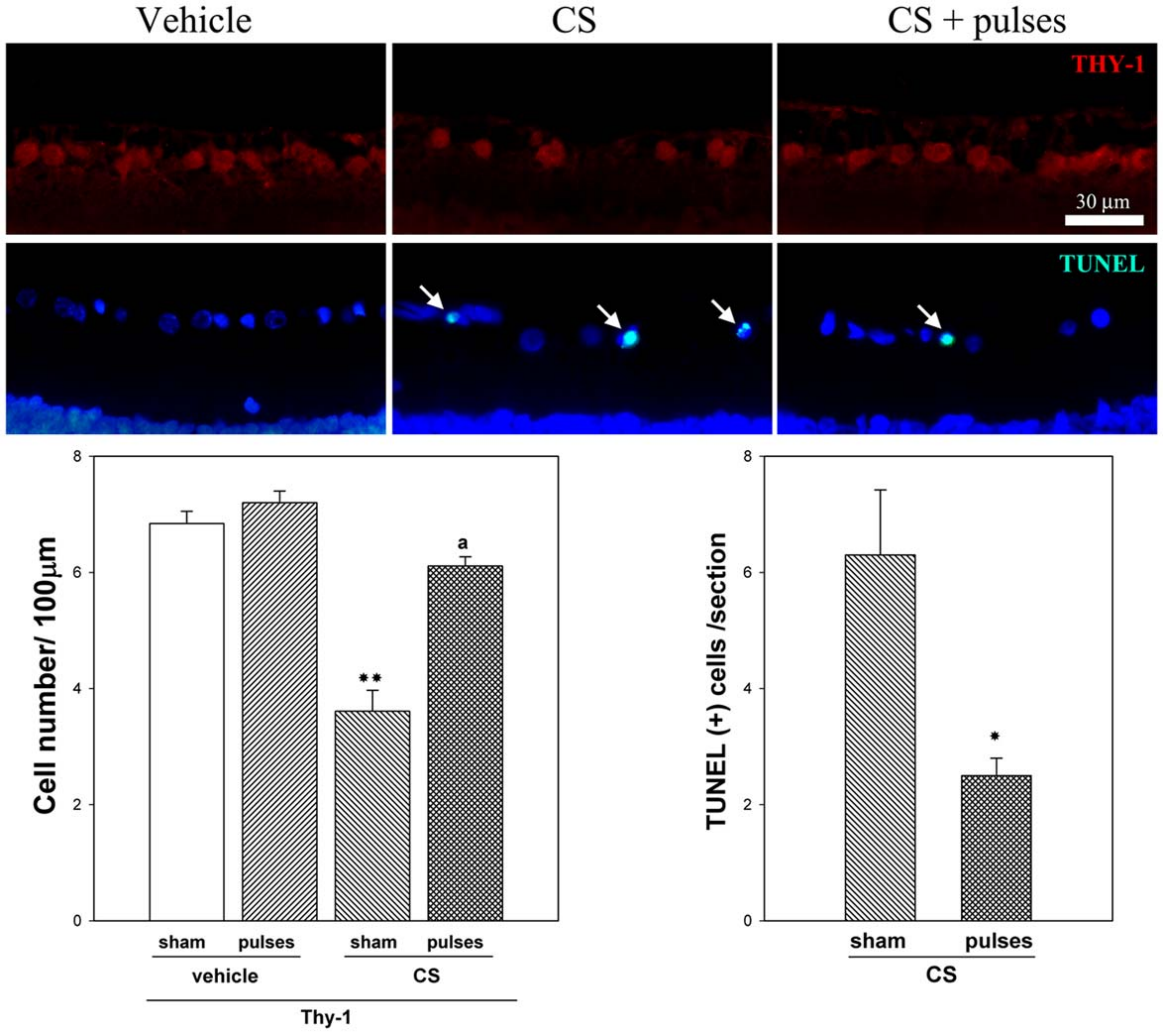
Thy-1 level and TUNEL assessment after 10 weeks of ocular hypertension. Representative photomicrographs showing Thy-1 immunofluorescence (upper panel) and TUNEL analysis (middle panel) from a vehicle-injected eye, and a hypertensive eye without or with pulses of ischemia. Note TUNEL^+^ cells in the GCL (arrows). Cell nuclei were counterstained with DAPI. Lower panel: The number of Thy-1 positive cells was significantly lower in hypertensive eyes without ischemia pulses than in vehicle- injected eyes, whereas the application of ischemia pulses in CS-injected eyes increased Thy-1 immunostaining. The number of TUNEL^+^ cells in the GCL was significantly higher in hypertensive eyes submitted to a sham procedure than in those submitted to ischemia pulses. No TUNEL^+^ cells were observed in vehicle-injected eyes submitted to ischemia pulses or sham procedure (not shown). Scale bar: 30 µm. Data are mean ± SEM (n = 5 eyes per group). For TUNEL analysis: * p<0.05 versus CS-injected eyes submitted to sham procedure, by Students t-test; For Thy-1 analysis: **p<0.01 versus vehicle injected eyes without ischemia pulses (sham), a: p<0.01, versus CS-injected eyes without ischemia pulses (sham), by Tukey's test.

The effect of ischemia pulses on histological alterations induced by ocular hypertension was examined. A morphometric analysis of retinal sections performed at 10 weeks of treatment with vehicle or CS revealed no differences in the total retina, IPL, INL, OPL and ONL thickness (data not shown), whereas a significant decrease in the number of cells in the GCL was observed in CS-treated eyes submitted to a sham procedure. Ischemia pulses prevented GCL cell loss, as shown in Figure 3 (upper panel). NeuN-positive cells in the retina from vehicle or CS-injected eyes without or with ischemia pulses were counted, and these sections were counterstained with the fluorescent nuclear stain DAPI (Figure 3, middle panel). A statistically significant increase in the number of H&E stained cells, NeuN positive cells, and DAPI-labeled cells in the GCL was observed in hypertensive eyes submitted to ischemia pulses, as compared with hypertensive eyes submitted to a sham procedure (Figure 3, lower panel). Ocular hypertension induced a significant decrease in the number of Thy-1 positive cells, whereas a significant preservation of Thy-1 positive cell number was observed in hypertensive eyes submitted to ischemia pulses, as shown in Figure 4. Apoptotic cell death was evaluated by the terminal deoxynucleotidyl transferase dUTP nick end labeling (TUNEL) method. A significant increase in the number of TUNEL^+^ cells in the GCL was observed in the retina from eyes injected with CS and submitted to a sham procedure, whereas ischemia pulses significantly decreased the number of apoptotic cells (Figure 4). No TUNEL^+^ cells were observed in retinas from eyes injected with vehicle and submitted to ischemia pulses or sham procedure (data not shown).

**Figure 5:**
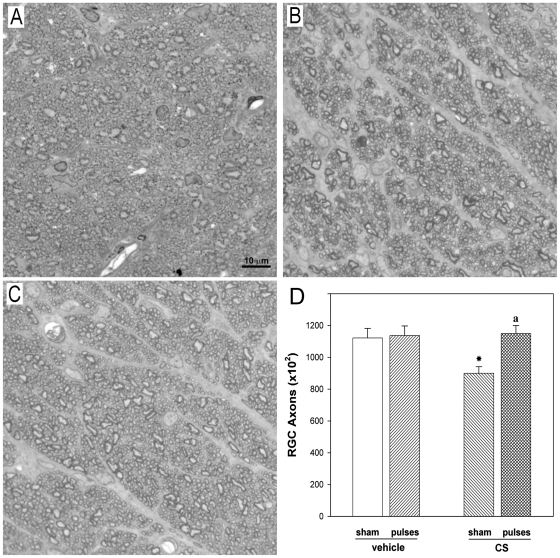
ONH sections from a control or a CS-treated eye without or with ischemia pulses. (A) Healthy, intact control optic nerve. Note the homogeneity of the staining. In vehicle-injected eyes, individual axons were generally uniform in shape, rounded and packed together tightly to form the fibers of the healthy nerve. In CS-treated eye without ischemia pulses (B) a less stained area indicates a nerve alteration. Disease in individual axons was characterized by axonal distention and distortion that resulted in a departure from the circular morphology of normal axons. In contrast, a conserved structure of the ONH was observed in the CS-treated eye with ischemia pulses (C). Toluidine blue. (D) Number of axons in eyes injected with vehicle or CS without or with ischemia pulses. A significant decrease in the axon number was observed in CS- injected eyes without ischemia pulses as compared with vehicle-injected eyes (sham), whereas ischemia pulses significantly preserved this parameter. Scale bar: 10 µm. Data are mean ± SEM (n = 5 eyes/group) *p<0.05 vehicle injected eyes without ischemia pulses (sham), a: p<0.05 versus CS-injected eyes without ischemia pulses (sham), by Tukey's test.

Axons in the ONH from vehicle-injected eyes were generally uniform in shape (rounded) and showed a variable size (Figure 5A). The ONH from eyes treated with CS for 10 weeks without ischemia pulses exhibited an overall loss of staining uniformity and integrity, showing distention and distortion that resulted in a departure from the circular morphology of normal axons (Figure 5B). The application of ischemia pulses significantly preserved ONH structure (Figure 5C). No changes in the transversal area of the optic nerve were observed among vehicle-injected and CS-injected eyes submitted to ischemia pulses or sham procedure (i.e. transversal area of the optic nerve was: 0.217±0.01; 0.213±0.02; 0.217± 0.02 and 0.216±0.01 mm^2^, for vehicle + sham, vehicle + pulses, CS + sham, CS + ischemia pulses, respectively), but a significant decrease in the axon number was observed in eyes injected with CS, which was prevented by ischemia pulses (Figure 5D).

**Figure 6:**
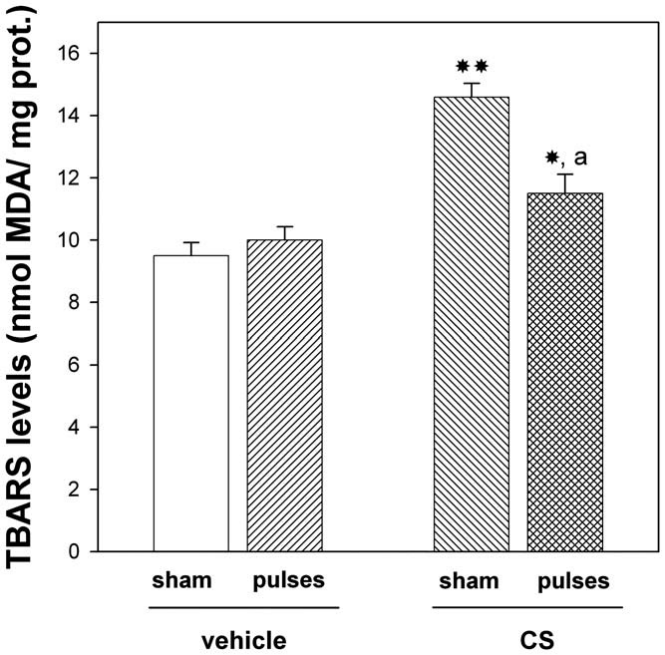
Retinal TBARS levels in animals injected with CS or vehicle, without o with ischemia pulses. This parameter was significantly higher in eyes injected with CS without ischemia pulses than in those injected with vehicle. The application of brief ischemia pulses in CS-injected significantly reversed the increase in retinal lipid peroxidation. Data are mean ± SEM (n = 12 eyes/group), * p<0.05, **p<0.01 vs. vehicle injected eyes without ischemia pulses (sham), a: p<0.05 vs. CS-injected eyes submitted to a sham procedure, by Tukey's test.

In order to analyze the involvement of oxidative stress in the protection induced by ischemia pulses, retinal lipid peroxidation was examined. Chronic injections of CS without ischemia pulses significantly increased thiobarbituric acid reactive substances (TBARS) levels, whereas the application of ischemia pulses significantly decreased this parameter, as shown in Figure 6.

## Discussion

The present results indicate that a weekly application of 5-min retinal ischemia pulses which showed no effect *per se*, abrogated functional and histological alterations induced by chronic ocular hypertension. Notably, the retinal protection induced by ischemia pulses was independent from ocular hypertension, as shown by the fact that ischemia pulses did not affect the increase in IOP induced by CS injections.

Human open angle glaucoma is a progressive optic neuropathy. In agreement, we have identified different stages in the experimental model of glaucoma induced by weekly injections of CS, that show the following characteristics: i) 3 weeks of ocular hypertension: no changes in the ERG, VEPs, and retinal morphology (i.e. **asymptomatic ocular hypertension**); ii) 6 weeks of ocular hypertension: decrease in ERG and VEPs, without histological changes (i.e. **moderated glaucoma)**; and iii) 10 weeks of ocular hypertension: further decrease in ERG and VEPs (vs. 6 weeks), and loss of RGCs and optic nerve fibers (i.e. **advanced glaucoma**) [1]. In order to assess whether the induction of ischemic tolerance was able to reduce glaucoma progression, the application of ischemia pulses started at 6 weeks of treatment with CS, a time point in which functional alterations are already evident.

Several observations support that some components of the flash ERG [19], [20] and VEPs [21], [22] can be affected in experimental models of glaucoma. The weekly application of ischemia pulses, which did not show any effect in control retinas, prevented the decrease in the ERG a- and b-wave and flash VEP N2-P2 amplitude induced by chronic ocular hypertension, supporting that the induction of ischemic tolerance not only preserved the retinal function, but also the activity of all cells in the pathway from photoreceptors to visual cortex, including RGCs and their axons.

In addition to RGCs, the GCL is comprised of a number of displaced amacrine cells. NeuN is a DNA-binding protein that identifies most mature neuronal populations, which has been used as a specific marker for RGCs [23]-[25], while Thy-1 is a surface glycoprotein uniquely expressed in RGCs. In the retina from eyes injected with CS without ischemia pulses, a significant loss of GCL cells, as shown by H&E and DAPI staining, and NeuN and Thy-1 immunohistochemistry was observed, without changes in IPL, INL, OPL, and ONL thickness. Ischemia pulses significantly reduced the effect of ocular hypertension on the number of cells in the GCL, as well on the number of apoptotic cells in the GCL. In addition, a significant decrease in the axon number was evident in hypertensive eyes without ischemia pulses, whereas ischemic conditioning significantly prevented the effect of ocular hypertension on ONH axon number.

Although the influence of duration and frequency of ischemia pulses on retinal protection deserves to be further examined, these results indicate that a significant restoration of retinal alterations provoked by chronic ocular hypertension can be achieved by the procedure performed herein.

Evidence has progressively accumulated to suggest that vascular insufficiency plays an important role in the pathogenesis of glaucomatous neuropathy, and a strong case has been made out to support the view that glaucoma may involve an ischemic-like insult to RGCs [6], [26]. IPC and PostC are highly effective strategies to protect the retina from an acute and deleterious ischemic episode. The precise mechanisms responsible for the retinal protection against glaucomatous damage induced by ischemia pulses remain to be established. Several common mechanisms have been involved in glaucomatous and ischemic damage. Oxidative stress, excitotoxicity, cell acidosis, inflammation, and others mechanisms acting in tandem are of considerable importance in both retinal ischemia and glaucoma. Among all retinal neurons, RGCs are most susceptible to ischemic and glaucomatous damage [27]. Several lines of evidence support that oxidative damage plays a major role in glaucoma pathogenesis [11], [28]. In that sense, we have demonstrated that ocular hypertension provokes a significant decrease in the retinal endogenous antioxidant defense system activity [11]. Moreover, retinal lipid peroxidation significantly increases in a time-of-hypertension-dependent manner [11]. The present results indicate that ocular hypertension induced by chronic injections of CS significantly increased retinal lipid peroxidation, and that induction of ischemic tolerance decreased this parameter. Thus, without excluding the activation of other transduction pathways associated with retinal IPC, such as adenosine, K_ATP_ channels, Protein Kinase C, heat shock proteins and NO [14], [29], [30] among others, and based on the fact that ischemia pulses abrogated the increase in lipid peroxidation, it is tempting to speculate that the induction of ischemic conditioning could behave as an antioxidant therapy.

Brief ischemia or hypoxia serve as prototypical conditioning stimuli; however, ischemic tolerance can be induced by exposing animals or cells to diverse type of endogenous or exogenous stimuli that are not necessarily hypoxic or ischemic in nature, such as hyperbaric oxygenation [31], or hyperthermia [32] among others. Recently, we have shown that a moderate inflammation induced by a single intravitreal injection of bacterial lipopolysaccharide provides retinal protection against ischemia/reperfusion injury [33]. In fact, IPC also induces retinal protection against a non-ischemic insult, such as light-induced injury [34]. It has been postulated that ischemic tolerance provokes the attenuation of broad categories of injury-inducing mechanisms, including excitotoxicity, ion/pH imbalance, oxidative and nitrosative stress, metabolic dysfunction, inflammation and, ultimately, necrotic and apoptotic cell death (reviewed in [15]). Several of these mechanisms have been involved in glaucomatous damage. Thus, although the present results do not necessarily support the involvement of retinal ischemia in glaucoma, they indicate that protection against glaucomatous damage can be achieved by the induction of ischemic tolerance.

Many exogenously delivered chemical preconditioning agents (for example, inflammatory cytokines, anaesthetics, and metabolic inhibitors) can also induce ischemic tolerance, raising the hope that in the future, IPC and PostC could be pharmacologically “mimicked” *in vivo* [15]. Therefore, the present results support that induction of ischemic tolerance could constitute a fertile avenue for the development of new therapeutic strategies in glaucoma treatment.

## Materials and Methods

### Ethics Statement

All animal procedures were in strict accordance with the ARVO Statement for the Use of Animals in Ophthalmic and Vision Research. The ethic committee of the School of Medicine, University of Buenos Aires (Institutional Committee for the Care and Use of Laboratory Animals, (CICUAL)) approved this study.

### Animals

Male *Wistar* rats (average weight 250±40 g) were housed in a standard animal room with food and water *ad libitum* under controlled conditions of humidity and temperature (21°±2°C). The room was lighted by fluorescent lights that were turned on and off automatically every 12 hours, with the lights (200 lux) on from 6.00 A.M. to 6.00 P.M. A total number of 84 animals were used for the experiments, distributed as follows: 10 animals bilaterally injected with vehicle (control) and 10 animals bilaterally injected with CS for IOP, ERG and VEP assessment at 10 weeks of treatment, 5 control and 5 CS-injected animals for histological studies, 5 animals of each group (control and CS) for the study of the ONH, and 12 control and 12 CS animals for TBARS assessment. Moreover, 10 control and 10 CS-injected animals were used for the IOP assessment at different time points, and 5 retinas per group from these animals were used for TUNEL, and Thy-1 analysis.

### Intracameral injections

**Figure 7:**
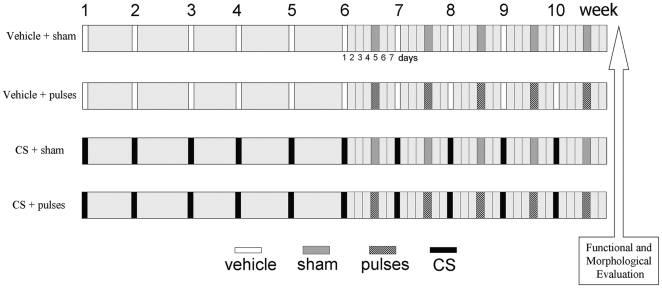
Experimental groups. Ischemia pulses or a sham procedure were applied 4 days after a once-a-week intracameral injection of vehicle or CS, starting after 6 weekly intracameral injections.

Rats were anesthetized with ketamine hydrochloride (150 mg/kg) and xylazine hydrochloride (2 mg/kg) administered intraperitoneally. CS was obtained from Sigma Chemical Co., St. Louis, MO, USA, catalog # C9819, isolated from bovine trachea. Using a Hamilton syringe with a 30-gauge needle, 20 µl of CS (0.4 g/ml in saline solution) were injected in both eyes, whereas control animals were bilaterally injected with saline solution (Figure 7). The eyes were focused under a Carl Zeiss (model OMNI MDU XY, Carl Zeiss, Oberkochen, Germany) surgical microscope with coaxial light. The needle moved through the corneoscleral limbus to the anterior chamber with the bevel down. When the tip of the bevel reached the anterior chamber, the liquid progressively increased the chamber's depth, separating the needle from the iris and avoiding needle-lens contact. Applications were made slowly but using a force sufficient to just empty the syringe content (adjusted to 20 µl). In the chronic protocol, injections were applied at the corneoscleral limbus beginning from hour 12 and changing the site of the next injection from hour to hour by rotating the head to achieve better access to the limbus. The injections and IOP assessments were performed after applying one drop of 0.5% proparacaine hydrochloride to each eye. Rats showing cataract (less than 4%) were excluded from the experiments. In addition, almost all the animals developed a localized corneal edema at the site of the injection that lasted less than 24 h. No differences in the incidence of these ocular complications were detected between CS and saline-injected eyes, and between eyes submitted to ischemia pulses and those submitted to a sham procedure.

### Ischemic conditioning protocol

Rats were assigned to different groups depicted in Figure 7. Ischemia pulses were applied in one eye from animals bilaterally injected with vehicle or CS, whereas the contralateral eye was cannulated without raising IOP (sham procedure). The selection of eyes that received ischemia pulses or sham treatment was performed at random. Before ischemia pulse or sham procedure application, IOP was assessed in both eyes from each animal, and the starting IOP values did not differ between both eyes from the same animal. Animals were anesthetized with ketamine hydrochloride (150 mg/kg) and xylazine hydrochloride (2 mg/kg) administered intraperitoneally. After topical instillation of proparacaine, the anterior chamber was cannulated with a 30-gauge needle connected to a pressurized bottle filled with sterile normal saline solution. Retinal ischemia was induced by increasing IOP to 120 mm Hg for exactly 5 min, as previously described [18]. With this maneuver, complete ocular ischemia was produced, characterized by the loss of electroretinogram (ERG) b-waves and the cessation of flow in retinal vessels, determined by funduscopic examination. Brief retinal ischemia was weekly applied, starting at 6 weeks of ocular hypertension and continuing until week 10. During ischemia pulses, animals were kept normothermic with heated blankets. Each ischemia pulse was applied 4 days after the injection of vehicle or CS.

### IOP assessments

A TonoPen XL (Mentor, Norwell, MA) tonometer was used to assess IOP in conscious, unsedated rats as previously described [1]. IOP determinations were assessed by operators who were blind with respect to the treatment applied to each eye. Animals were wrapped in a small towel and held gently, with one operator holding the animal and another making the readings. Five IOP readings were obtained from each eye by using firm contact with the cornea and omitting readings obtained as the instrument was removed from the eye. The mean of these readings was recorded as the IOP for this eye. Mean values from each rat were averaged, and the resultant mean value was used to compute the groups mean IOP ± SE. Differences among reading were less than 10% (standard error).

### Electroretinography

Electroretinographic activity was registered as previously described [1], [18]. Briefly, after 6 h of dark adaptation, rats were anesthetized under dim red illumination. Phenylephrine hydrochloride and tropicamide were used to dilate the pupils, and the cornea was intermittently irrigated with balanced salt solution to maintain the baseline recording and to prevent keratopathy. Rats were placed facing the stimulus at a distance of 20 cm. All recordings were completed within 20 min and animals were kept warm during and after the procedure. A reference electrode was placed through the ear, a grounding electrode was attached to the tail, and a gold electrode was placed in contact with the central cornea. A 15W red light was used to enable accurate electrode placement. This maneuver did not significantly affect dark adaptation and was switched off during the electrophysiological recordings. ERGs were recorded from both eyes simultaneously and ten responses to flashes of unattenuated white light (5 ms, 0.2 Hz) from a photic stimulator (light-emitting diodes) set at maximum brightness (0.95 log cd.s/m^2^ without filter) were amplified, filtered (1.5-Hz low-pass filter, 1000 high-pass filter, notch activated) and averaged (Akonic BIO-PC, Argentina). The a-wave was measured as the difference in amplitude between the recording at onset and the trough of the negative deflection and the b-wave amplitude was measured from the trough of the a-wave to the peak of the b-wave. Electrophysiological responses were averaged for each run. Runs were repeated 3 times with 5 min-intervals to confirm consistency and the mean of these 3 runs was used for subsequent analysis. The mean peak latencies and peak-to-peak amplitudes of the responses from each group of rats were compared.

### Flash visual evoked potentials

Scotopic flash visual evoked potentials (VEPs) were registered as previously described [1]. For this purpose, two stainless steel electrodes were surgically placed 4 mm lateral to the interhemispheric fissure and 5,6 mm behind bregma (active electrode). Reference electrodes were placed 2 mm lateral to the midline and 2 mm before bregma. A ground electrode was placed in the animal tail. Both electrodes were isolated and fixed with dental acrylic and the skin was sutured with nylon 5/0. Five days after electrode implantation, VEPs were assessed as follows: after 6 h of dark adaptation, rats were anaesthetized, pupils were dilated and the cornea was intermittently irrigated as previously described, under dim red illumination. All recordings were completed within 20 min of the induction of anaesthesia and animals were kept warm during and after the procedure. Each eye was registered individually, occluding the contralateral eye with black carbon paper and cotton, and a 70 stimuli average was registered. Eyes were stimulated with unattenuated white light (1 Hz) from a photic stimulator (light-emitting diodes) set at maximum brightness (0.95 log cd.s/m^2^) were amplified, filtered (0.5-Hz low-pass filter, 100 high-pass filter, notch activated) and averaged (Akonic BIO-PC, Akonic, Buenos Aires, Argentina). The amplitude between the N2 deflection and the P2 peak was assessed, and the N2 latency was measured from de onset to the second negative peak.

### Light Microscopy

Eyes were enucleated after anesthetic overdose and immersed immediately in a fixative containing 4% paraformaldehyde in 0.1 M phosphate buffer (pH 7.2) for 1 h. The nictitans membrane was maintained in each eye to facilitate orientation. The cornea and the lens were carefully removed, and the posterior portions were fixed for an additional 12 h- period in the same fixative. A cross section of the optic nerve from vehicle and CS-treated eyes was removed 1.5 mm posterior to the globe and postfixed in 1% osmium tetroxide in phosphate buffer. Nerves were processed into epoxy resin, sectioned at 1 µm, and stained with 1% toluidine blue. Eyecups were dehydrated in an alcohol series, and embedded in paraffin. Sections (5 µm thick) were cut along the horizontal meridian through the ONH and stained with hematoxylin and eosin (H&E) or used for immunofluorescence and TUNEL analysis.

### NeuN and Thy-1 immunofluorescence

Antigen retrieval was performed by heating (90°C) slices for 30 min in citrate buffer and then preincubated with 2% normal horse serum, 0.1% bovine serum albumin, and 0.4% Triton X-100 in 0.01 M phosphate-buffered saline for 1 h. The sections were then incubated overnight at 4°C with a mouse monoclonal anti-NeuN antibody (1∶120; Millipore) or anti-Thy-1 antibody (1∶1000 Millipore). An anti-mouse secondary antibody conjugated to Alexa Fluor 568 (1∶500; Molecular Probes) was used. After immunostaining, the sections were mounted with antifade medium with the fluorescent dye DAPI (Vector Laboratories). Some sections were treated without the primary antibodies to confirm specificity. An Olympus BX50 microscope (Olympus, Tokyo, Japan) was used for microscopic observations. Comparative digital images from different samples were grabbed using identical time exposition, brightness, and contrast settings.

### Image analysis

Microscopic images were digitally captured with a Nikon Eclipse E400 microscope (illumination: 6-V halogen lamp, 20W, equipped with a stabilized light source) attached to a digital camera (Coolpix s10; Nikon). The digitalized images were transferred to a Scion Image for Windows analysis system (Scion Corporation Beta 4.0.2).

Retinal morphometry was evaluated as described by Takahata et al., (2003) [35], with minor modifications. Three sections were randomly selected from each eye. Nine microscopic images at 1 mm from the temporal edge of the optic disc were digitally analyzed. The light microscope was adjusted to level 4 and a 40x CF E achromat objective was used. The thickness (in µm) of the inner plexiform layer (IPL), inner nuclear layer (INL), outer plexiform layer (OPL), outer nuclear layer (ONL), and total retina was measured. The number of cells in the ganglion cell layer (GCL) was expressed as cells per 100 µm. For each eye, results obtained from three separate sections were averaged and the mean of 5 eyes was recorded as the representative value for each group. No attempt was made to distinguish cell types in the GCL for enumeration of cell number. The morphometric analysis was performed by observers masked to the protocol used in each eye.

### TUNEL analysis

For DNA fragmentation of cells undergoing apoptosis, the ApopTag® Fluorecein In Situ Apoptosis Detection Kit (S7110, Chemicon, CA, USA) was used according to manufacturer's instructions. For each retinal section, the number of TUNEL^+^ cells along the entire retina was calculated. For each eye, results obtained from four separate sections were averaged and the mean of five eyes was recorded as the representative value for each group.

### Optic nerve morphometry

Optic nerve axon counting was performed as previously described [1]. To measure axon density, images were captured with a 100X achromat objective from 5 spaced nerve regions. Images were converted to 8-bits grey scale and a manual threshold value, first determined by visual examination, was constantly applied. Finally, images were converted to a binary form. The number of axons counted in 5 images from each nerve was approximately 10% of the total optic nerve area. The counting process was performed by observers masked to the protocol used in each nerve.

### Measurement of TBARS levels

TBARS levels in retinal tissue were analyzed as previously described [11]. Retinas were homogenized in 15 mM potassium buffer plus 60 mM KCl, pH 7.2. The homogenate (300 µl) was mixed with 75 µl 10% SDS and 1.4 ml of 0.8% thiobarbituric acid dissolved in 10% acetic acid (pH 3.5). This solution was heated to 100°C for 60 min. After cooling, the flocculent precipitate was removed by centrifugation at 3200 x g for 10 min. After addition of 1.0 ml water and 5.0 ml of n-butanol-pyridine mixture (15∶1, vol/vol), the mixture was vigorously shaken and centrifuged at 2000 x g for 15 min. The absorbance of the organic layer was measured at an emission wavelength of 553 nm by using an excitation wavelength of 515 nm with a Jasco FP 770 fluorescence spectrophotometer (Japan Spectroscopic Co. Ltd. Tokyo, Japan). The range of the standard curves of malondialdehyde bis-dimethyl acetal (MDA) was 10–2000 pmol. Results were expressed as nanomol MDA per mg of protein.

Protein content was determined by the method of Lowry et al., (1951) [36], using bovine serum albumin as the standard.

### Statistical analysis

Statistical analysis of results was made by a Student's t-test or by a two-way analysis of variance (ANOVA) followed by Student's t-test or Dunnett's test, as stated.
